# Longitudinal multidiversity pattern and the environmental drivers of riparian bird communities along submontane rivers of Changbai Mountains, China

**DOI:** 10.1002/ece3.9542

**Published:** 2022-11-27

**Authors:** Wenyu Xu, Ye Gong, Lin Wang, Jiyuan Yao, Haitao Wang

**Affiliations:** ^1^ Jilin Engineering Laboratory for Avian Ecology and Conservation Genetics, School of Life Sciences Northeast Normal University Changchun China; ^2^ Jilin Key Laboratory of Animal Resource Conservation and Utilization Northeast Normal University Changchun China; ^3^ National Demonstration Center for Biological Experimental Teaching School of Life Sciences Northeast Normal University Changchun China; ^4^ Northeast Institute of Geography and Agroecology Chinese Academy of Sciences Changchun China; ^5^ Animal's Scientific and Technological Institute Agricultural University of Jilin Changchun China

**Keywords:** bird diversity, Changbai Mountain, environmental indicators, riparian birds, submontane rivers

## Abstract

Riparian zones are biodiversity hotspots in montane ecosystems and are of critical conservation concern. However, studies on longitudinal diversity patterns and environmental drivers have been restricted to aquatic fauna, while the animals that rely on both river and riparian resources have been of much less concern. Here, we examined the multifaceted diversity distribution of riparian birds along longitudinal gradients and analyzed the importance of environmental factors in shaping these patterns in the Changbai Mountains. Hump‐shaped relationships between elevation and taxonomic, phylogenetic, and functional diversity, as well as with the conservation value index, were evident along each of the classic submontane rivers. Forest cover, vegetation height variation, and land cover patches positively affected the taxonomic diversity indices. In addition to the species richness, fluvial geomorphology variables (river sinuosity and gravel bar) were significantly related to the phylogenetic diversity. However, there was no statistical evidence for a relationship between functional diversity and the environmental variables examined. This study emphasized the necessity of including multiple diversity measures beyond taxonomic diversity and demonstrated the importance of both terrestrial and aquatic components in shaping the multifaceted biodiversity pattern of riparian organisms living in riparian zones. The results suggested that conservation priority should be given to both rivers and banks in the middle reaches and that riparian birds could be good candidate indicators of environmental change in the submontane river‐forest ecotone.

## Graphical abstract

We examined the multifaceted diversity distribution of riparian birds along longitudinal gradients and analyzed the importance of environmental factors in shaping these patterns in the Changbai Mountains. We found the similar hump‐shaped distribution of multiple diversity in riparian bird communities. Forest cover, vegetation height variation, and land cover patches were significantly related to biodiversity. The results suggested that conservation priority should be given to both rivers and banks in the middle reaches.

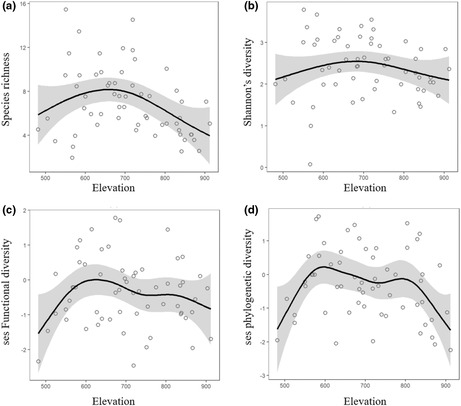

## 1 INTRODUCTION

Riparian zones are regarded as a potential focal point of biodiversity in montane ecosystems (Naiman et al., 1993; Palmer & Bennett, 2006), offering specific habitats and refuges for many taxonomic groups, including invertebrates, amphibians, fishes, and birds (Randall et al., 1996; Smiley et al., 2009; Steward et al., 2022). Several studies have been demonstrated that biodiversity is not evenly distributed within riparian zones, but changing with spatial gradients (Finn & Poff, 2005; Zhang et al., 2020). According to the River Continuum Concept, the longitudinal (upstream–downstream) biodiversity pattern forms in response to continuous changes in environmental conditions (RCC; Ward, 1989; Ward, 1998; Vannote et al., 1980). The mid‐peak distribution of species richness in various aquatic taxa along rivers has been commonly reported, such as in aquatic macroinvertebrates (Abdelsalam, 2012), mussels (Begley & Krebs, 2017), and fish (Zhang et al., 2020). In addition, the species richness of riparian organisms, when driven by particular environmental factors along mountainous rivers, can monotonically increase (Berrahou et al., 2001) or present U‐shaped distributions (Zhang et al., 2020).

Aquatic and/or terrestrial attributes have been found to influence longitudinal patterns of biodiversity along riparian zones (Corenblit et al., 2014; Royan et al., 2014). Specifically, river morphology and water physicochemical parmeters are regarded as key drivers in the longitudinal patterns of aquatic organisms (Usseglio‐Polatera & Beisel, 2002), and riverine landscape attributes, e.g., forest areas and vegetation compositions, are considered major actors in the longitudinal patterns of terrestrial organisms (Rakotomalala & Goodman, 2010). For taxa relying on both river and riparian resources, terrestrial and aquatic elements are assumed to jointly shape their longitudinal distribution patterns (Cubley et al., 2020). However, the drivers of longitudinal patterns in these taxa have been less frequently explored.

As a large group of species inhabiting submontane riparian zones, bird species are sensitive to riparian ecological conditions, and the diversity of bird guilds was usually used as a measurable target for ecosystem conservation and restoration by many conservation scientists, land managers and policymakers (Canterbury et al., 2000; Fleishman et al., 2003). While longitudinal patterns in bird communities both including river and land‐dwelling species along submontane riparian zones have been scarcely reported (Sinha et al., 2022). This situation is surprising given that birds are typically the most nutrient‐rich and dominant consumer group, utilizing both river channels and adjacent habitats during part, or all, of their lifecycles in submontane riparian zones.

The Changbai Mountains contain continuous forests embedded with classic submontane riparian zones and have upper‐middle‐lower reaches with obvious hydrogeomorphological characteristics. This region has been threatened by human activities (e.g., tourism and farming) due to rapid human development (Qi et al., 2018). However, the longitudinal patterns of biodiversity and the corresponding inferred drivers are not well understood (but see. Li et al., 2022; Ping et al., 2017). Most studies on species longitudinal distribution patterns rely on species richness due to its simplicity and convenience. However, measures of traditional biodiversity metrics such as species richness alone are relatively information poor. In fact, there were inconsistent relationships in evolutionarily independent and ecologically equivalent among species (Swenson, 2011). Phylogenetic and functional diversity (PD and FD) could use as alternative axes to compensate for inadequate biodiversity in evolutionary and functional terms. Comparisons of facets of biodiversity can offer complementary information and improve our understanding of the mechanisms underlying biodiversity patterns (Ding et al., 2021; He et al., 2018). Here, we explored the longitudinal patterns of multiple dimensions of diversity in bird communities and assessed the effect of environmental predictors in determining the bird community distribution patterns in the submontane rivers of the Changbai Mountains in northeast China. Specifically, we described the distribution patterns of multiple bird diversity along submontane riparian zones and assessed the ability of environmental variables to explain longitudinal patterns of multiple diversity in this study.

## 2 METHODS

### 2.1 Study area and sampling sites

**FIGURE 1:**
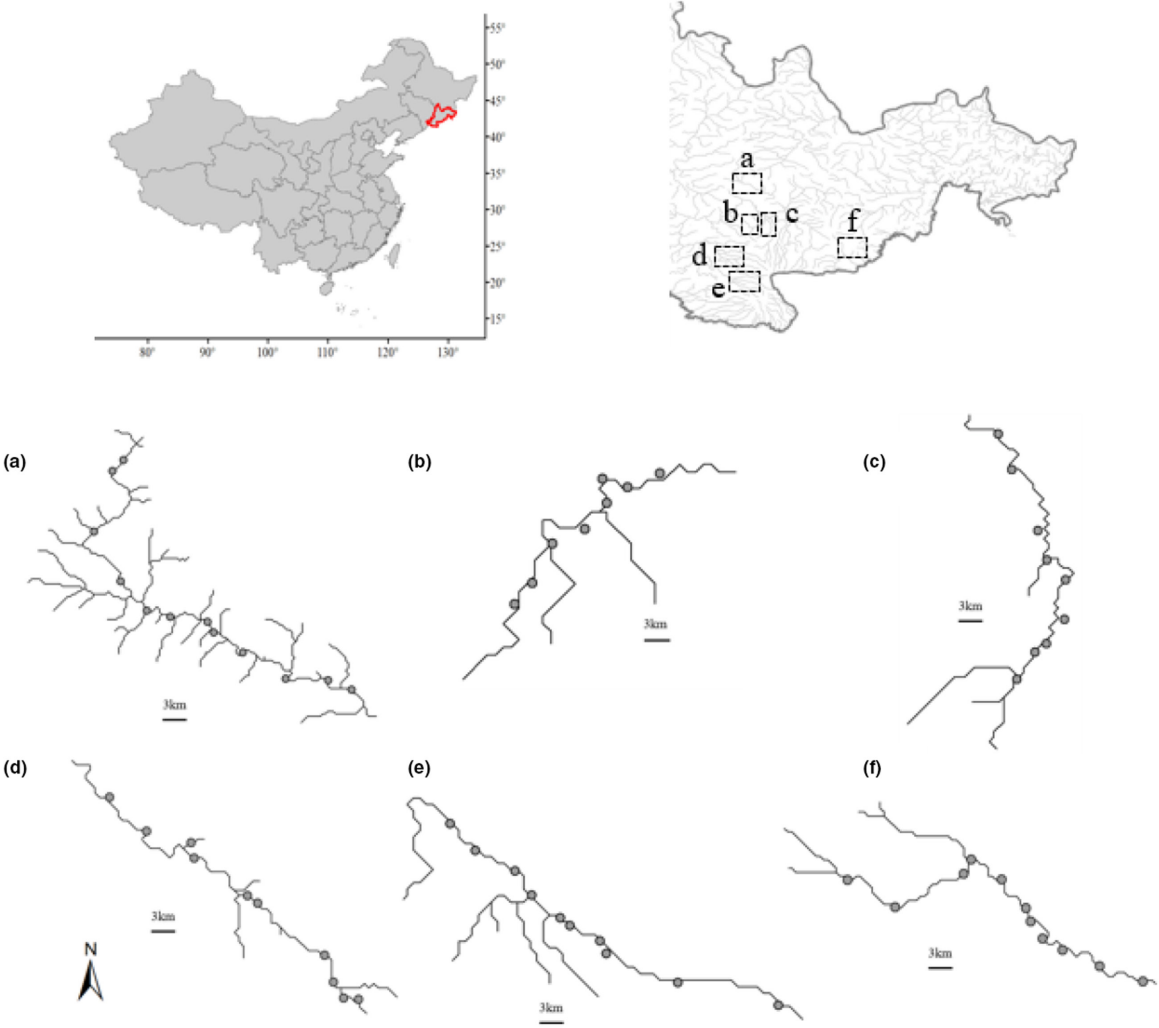
Locations of study submontane valleys and sample sites. Upper left inset locates Jilin Province in the Northeast China. Upper right inset shows specific locations of the six study submontane valleys.

This study was conducted at six classic submontane rivers in the Changbai Mountain area (41°51′‐43°3′ N, 127°32′‐129°0′ E; Figure 1): the Fuer River, the Lushui River, the Toudaobai River, the Songjiang River, the Manjiang River, and the Damalu River. The straight‐line distance between six rivers from 20 to 120 km. The rivers are characterized by naturally braided channels with many gravel alluvia, clay scarps, and forested banks. This region belongs to the temperate continental monsoon climate. The annual average temperature and precipitation are approximately 3.8°C and 680 mm, respectively. We chose a series of 8–12 sampling sites (481 to 912 masl) along each river (60 sampling sites in total) based on the elevation change rates (4.2–6.0) so that sites were as similar among streams as possible with respect to elevation. Finally, 2–4 sampling points were distributed in the upper, middle, and lower reaches of each river respectively, and the distance between adjacent points was from 2 to 9 km.

### 2.2 Bird survey

We conducted bird point‐count surveys during June 2021, during the peak of the bird breeding season (Bibby et al., 2000). The sampling sites were visited twice by two experienced ornithologists standing at the land–water interface and recording all birds seen or heard in 10 min within a 50‐m radius. The average river widths ranged between 12 and 21 m, meaning that the detection radius encompassed both the neighboring terrestrial area and the river. All field surveys were carried out on clear days within 4 h after sunrise in the absence of snow, rain, or strong winds.

### 2.3 Environmental data

In our study, both aquatic and terrestrial habitat attributes derived from different scales were considered to explain the biodiversity variations in bird communities living in riparian habitats. As environmental variables within microhabitats were found to impact birds diversity (e.g. Han et al., 2020; Hanle et al., 2020), three 5 × 5‐m quadrats were randomly selected at each sampling site to measure the microhabitat characteristics within each 50‐m radius of bird counting. For each quadrat, the proportions of trees, shrubs, herbs, bare lands, and plant canopies were visually estimated. In addition, the heights of trees, shrubs, and herbs were recorded with a laser rangefinder (Nikon Laser‐800) and tape line, and the vegetation height variability was determined by calculating the standard deviations of the heights of these three types of vegetation (Bae et al., 2018) to reflect the vertical heterogeneity in the vegetation structure. Riparian forests with higher vertical heterogeneity were predicted to host more diverse bird communities due to the stratification of microhabitats and resources (Parker & Brown, 2000). We further averaged values from the three sampling quadrates for each point.

In landscape scale, we collected predictors that were related to the ecological requirements of riparian birds within a 200 × 200‐m rectangle around each sampling site through visual interpretation from Landsat satellite imagery. These variables included the average river width, areas of gravel bars, and channel sinuosity (the ratio of the distance between two points along the channel and the straight line distance between these points). Habitat complexity is regarded as a critical factor in determining bird species richness and habitat selection (Foster et al., 2011; Hurlbert, 2004). We calculated the Shannon diversity and the number of land cover patches to represent habitat complexity according to 30‐m‐resolution raster data in which land cover types were divided into 20 categories (such as cropland, wetland, mixed forest, etc.; see Table S1; http://www.geodata.cn). All analyses related to landscape variables were performed using ArcGIS 10.2 (ESRI, 2014).

### 2.4 Multifaceted diversity

Species richness was measured as the number of bird species recorded at each sampling site. Alpha diversity was calculated with the Shannon–Weiner index (Hill, 1973). Functional diversity was measured as functional richness (FRic), which calculates the volume of the functional space occupied by the community (Cornwell et al., 2006). We determined the functional diversity by combining the relative bird abundance data with six key biological traits (e.g. trophic level, body mass, and nest site; see Table S2). All these traits are linked with species habitat selection, resource requirements, or reproduction. Based on these functional traits, we computed abundance‐weighted functional diversity using the function “dbFD” in the R package “FD” (Laliberte & Legendre, 2010). To calculate bird PD, we used the “Phylogeny subsets” tool from “Bird Tree” (http://birdtree.org) to construct a phylogenetic tree with 47 bird species. PD was then calculated in picante package, which reflected the sum of branch lengths of the phylogeny connecting all species within a given community (Kembel et al., 2010). In addition, we tested for the phylogenetic signal of each trait for each mountain system by *K*‐statistic using functions of the “phytools” package in R (Blomberg et al., 2003; Losos, 2008). In consideration of the high correlation with FD, PD, and species richness (FD: *r* = .58. *p* < .01; PD: *r* = .89, *p* < .01), we calculated the standardized effect size (*SES*) of FD and PD using null models to control for the effects of species richness (Petchey, 2004). *Ses* was calculated as (observed diversity–mean of randomized diversity)/SD of randomized diversity. Negative or positive values of *ses* indicate a lower or higher observation value than expected by chance. We randomized the abundance matrix with 1000 replicates and kept species richness constant in each band. FD and PD both had low Pearson correlation coefficients (|*r*| < .10) with species richness after the standardized processing step, while species richness and Shannon diversity were highly correlated. In addition, we calculated the conservation value index (CVI) as CVI = sum [log(*a*_i_ + *1*) × *e*_i_] for each sampling location to highlight the areas that needed the most attention and protection in submontane riparian zones (Pons et al., 2003), where *a*_*i*_ and *e*_*i*_ represents species abundance and conservation values respectively. Conservation values were attributed based on the List of Key Protected Wild Animals in China as follows: one for unthreatened species, four for class‐II protected species, and eight for class‐I protected species.

### 2.5 Statistical analyses

Prior to the analysis, the variance inflation factor (VIF) analyses were performed of all environmental predictors to limit multicollinearity in the car package (Hijmans & Van Etten, 2012). We removed explanatory variables with VIF values <4 by a stepwise approach, such that only nine variables remained for use as model predictors (Table 1).

**TABLE 1:** Variables filtered by variance inflation factor (VIF) for generalized additive mixed model (GLM) analysis.

Variables	Description	Abbreviation
Forest proportion	The proportion of forests at each sample site	Forest %
Shrub proportion	The proportion of shrubs at each sample site	Shrub %
Herb proportion	The proportion of herbs at each sample site	Herb %
Bared land proportion	The proportion of bared land at each sample site	Bared %
Land cover patches	Numbers of land cover patches at each 200*200 m quadrats	Lnum
Bar areas	Areas of river bars at each 200*200 m quadrats	Bar
River width	The average river width at each 200*200 m quadrats	Width
River sinuosity	The calculated channel sinuosity at each 200*200 m quadrats	Sinuosity
Vegetation height variability	The standard deviation of the average height of trees, shrubs, and herbs represents the vegetation vertical structure variability at each sample site	*H*_SD_

The distributions of bird species richness and multiple diversity dimensions along the longitudinal gradient were examined using generalized additive mixed models (GAMMs) with Gaussian error distribution in the mgcv package (Wood, 2011). The GAMMs were used to analyze the relationship between multiple diversity dimensions of bird assemblages and environmental predictors with lme4 package (Bates et al., 2014). We fit a GAMM with species richness and multiple diversity respectively as a responsible variable, elevation as a smoothed predictor variable, river direction, and river ID as a random variable. We calculated the ΔAICc and Akaike's weights (ωi) and then select the most or equally plausible models (ΔAICc ≤ 2) from our candidate sets based on ranked AICc scores with the dredge function in MuMln package (Burnham & Anderson, 2002). Finally, we combined all models with ΔAICc of ≤2 into a single, averaged model and calculated the model‐averaged coefficients, as well as the relative importance for each variable. The model‐averaged parameters will reduce the model selection bias and accounts for the model selection uncertainty (Lukacs et al., 2009). All analyses were conducted in R software (R Core Team, 2014).

## 3 RESULTS

We recorded a total of 875 individuals from 65 species across the 60‐point counts, including 12 species of river‐dwelling birds and 53 species of forest‐dwelling birds (Table S2). The gray wagtail (*Motacilla cinerea*) showed the highest occurrence in all sampling points (32 out of 60; 53.3%) followed by Pallas's leaf warbler (*Phylloscopus proregulus*; 51.7%). To reduce the potential biases in survey data, we restricted our analyses to those 47 species known to use riparian zones as their main breeding habitat and excluded 18 species with the low occurrence, detected at less than three sites. The species richness of the 47 remaining species was 7.18 ± 3.09 (range of 1 to 15); the Shannon diversity was 2.41 ± 0.72 (range of 0 to 3.75); sesFD was −0.40 ± 0.13 (range of −2.55 to 1.79); sesPD was −0.28 ± 0.13 (range of −2.25 to 1.73); and CVI was 4.07 ± 2.85 (range of 0.3 to 11.62). Phylogenetic signal of 53 riparian bird species was listed in Table S3, with body mass (*K* = 1.91, *p* < .001) showing a significant phylogenetic signal in trait data that departs from a model of trait evolution under Brownian motion, and the other 5 traits have a weak phylogenetic signal.

**FIGURE 2:**
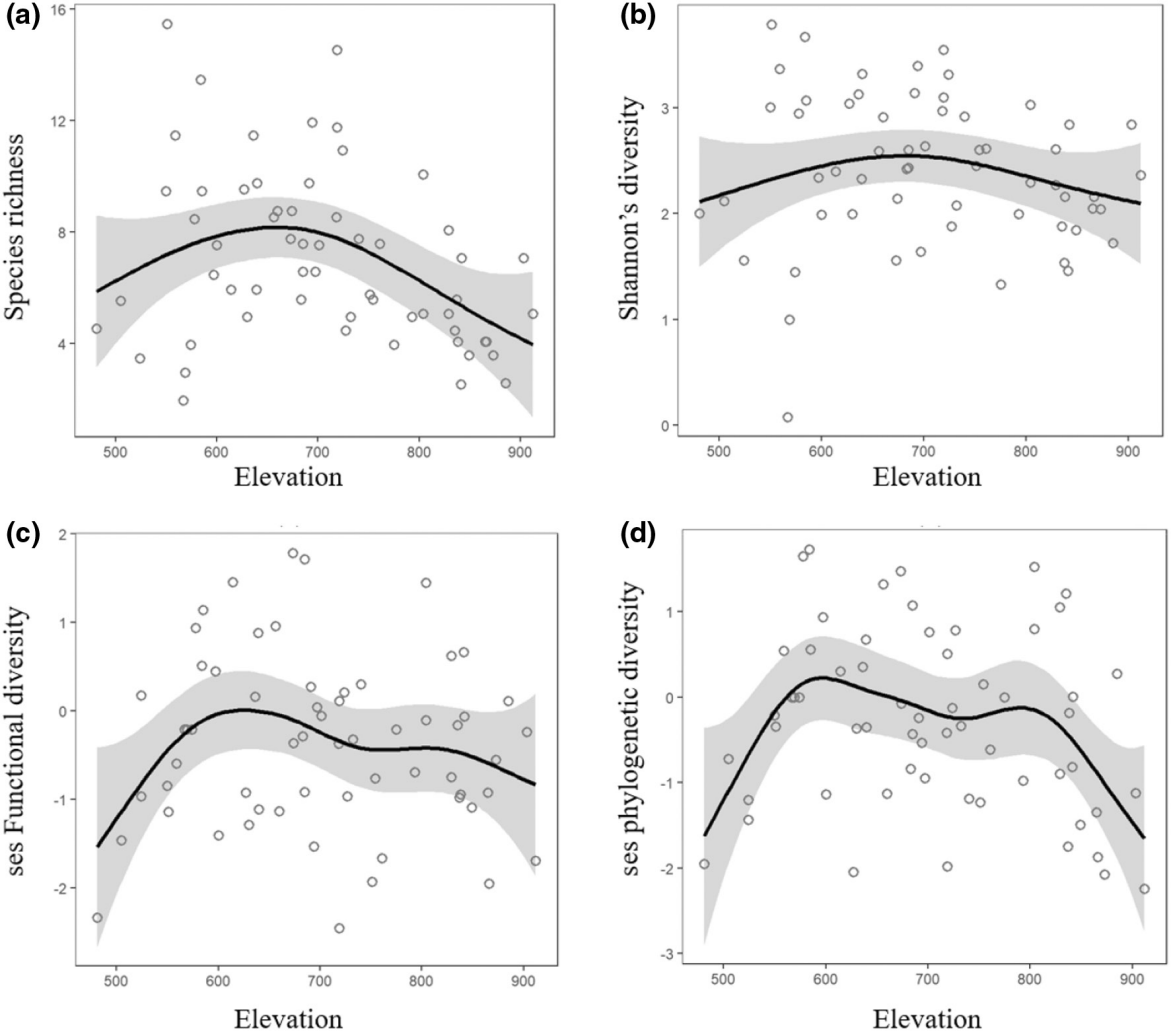
Patterns of (a) bird species richness and (b) Shannon diversity, (c) functional diversity, (d) phylogenetic diversity along the longitudinal gradient at six submontane river valleys of Changbai Mountain areas.

The bird species richness (9 species) and the Shannon diversity (2.68) showed a hump‐shaped pattern at intermediate elevations (Figure 2a,b) of approximately 700 masl. The bird FD and PD values showed the highest levels at relatively low elevations, with maxima at approximately 605 masl, followed by monotonic decreases as high‐elevation regions were sampled (Figure 2c,d).

**FIGURE 3:**
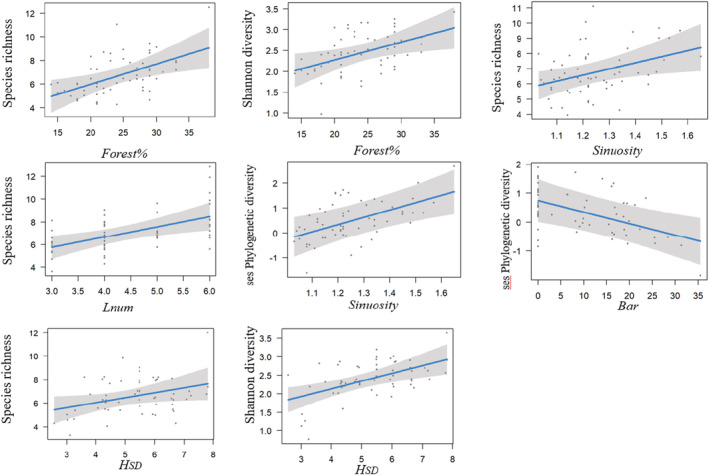
Significant relationships between multidimensional diversity and environmental predictors fitted by Bayesian generalized additive mixed models. Solid line represents the mean values, and the gray area represents 95% confidence intervals.

Based on model averaging, the bird species richness increased with forest cover, land cover patches, river sinuosity and *H*_SD_. (Table 2, Figure 3). Shannon diversity exhibited a similar response pattern as species richness (Table 2, Figure 3). We did not find any significant relationships between functional diversity and environmental predictors used in this study. (Table 2, Figure 3). Phylogenetic diversity was negatively correlated with the areas of river bars but positively correlated with river sinuosity (Table 2, Figure 3).

**TABLE 2:** Model‐averaged parameter estimates, SE, and *p* values explaining the relationships between bird species richness, multifaceted diversity, and environmental predictors.

	Parameter	Estimate	SE	*p*‐value
Species richness	(Intercept)	−0.71	2.97	.02
	Forest%	**0.17**	**0.06**	**<.01** ^***^
	Herb%	−0.07	0.04	.13
	Bared%	0.01	0.02	.67
	*H*_SD_	**0.40**	**0.19**	.04*
	Lnum	**0.91**	**0.28**	**<.01** ^***^
	Bar	0.01	0.01	.71
	Sinuosity	**4.50**	**1.59**	**<.01** ^***^
	Width	−0.01	0.01	.78
Shannon diversity	(Intercept)	−1.11	0.59	.06
	Forest%	**0.05**	**0.01**	**<.01** ^***^
	Barred%	−0.01	0.01	.84
	*H*_SD_	**0.19**	**0.06**	**<.01** ^***^
	Lnum	0.11	0.10	.25
	Sinuosity	0.54	0.53	.31
Functional diversity	(Intercept)	−2.1	1.35	.12
	Forest%	−0.01	0.01	.79
	Shrub%	−0.01	0.02	.63
	Barred%	0.01	0.01	.89
	Lnum	−0.08	0.15	.60
	Bar	−0.02	0.02	.29
	Sinuosity	1.43	1.13	.21
	Width	0.01	0.01	.90
Phylogenetic diversity	(Intercept)	−2.81	1.12	.01
	Shrub%	−0.01	0.02	.54
	Herb%	0.01	0.01	.83
	Bared%	−0.01	0.01	.59
	*H*_SD_	0.03	0.07	.70
	Lnum	−0.07	0.14	.61
	Bar	**−0.03**	**0.01**	.04*
	Sinuosity	**2.66**	**0.90**	**<.01** ^**^

*Note*: *, ** and *** represent significant level.

## 4 DISCUSSION

In the present study, we examined bird community distribution patterns along longitudinal gradients and identified the key habitat attributes that drive multiple dimensions of bird community diversity in submontane riparian zones in the Changbai Mountains. The longitudinal gradients of the physically similar Changbai Mountain rivers were accompanied by a relatively predictable spatial arrangement of riparian birds that showed a hump‐shaped distribution, in alignment with the findings of the mid‐peak pattern in some other riparian organisms (Li et al., 2018; Torgersen et al., 2006). In contrast with most studies on the environmental factors driving the longitudinal patterns of riparian fauna (Wang et al., 2019), our study found that the longitudinal pattern of bird communities was driven by combined aquatic and terrestrial elements. The complexity of ecological systems was better described by focusing on multiple facets of bird diversity.

### 4.1 The longitudinal biodiversity pattern of bird communities

The mid‐peaked pattern is common and long‐noted in river ecology (Carvalho‐Rocha et al., 2020; Ding et al., 2021; Wu et al., 2012). This study added to the evidences by confirming that the longitudinal biodiversity pattern exhibited in riparian bird communities. In consistence, a hump‐shaped distribution of CVI (an index used to help conservationists prioritize action to where it is most needed; Terraube et al., 2016) was found along the submontane rivers. The higher CVI was mainly related to the occurrence of the protected waterbirds (Scaly‐sided Merganser *Mergus squamatus and* Mandarin duck *Aix galericulata*), which could potentially indicate high biodiversity and classic submontane riparian habitats (Xu et al., 2020). Those longitudinal patterns highlight the high conservation value of bird community, as well as essential habitat characteristics, in midstream.

A variety of hypotheses related to climate, evolutionary history, space, biotic processes, and the combinations and/or interactions of these factors have been proposed to explain this mid‐peaked distribution pattern (McCain, 2007; Rahbek, 1997; Zhang et al., 2020). Nevertheless, shared environmental response was found to be the dominant community assembly rule in riparian birds (Royan et al., 2015). Based on the RCC, riparian habitat diversity and complexity often increase along a longitudinal gradient to reach a maximum in middle reaches of rivers (Resh et al., 1988; Vannote et al., 1980). Habitat complexity has been widely proven to have a positive relationship with biodiversity due to increased microhabitat availability (Angermeier & Schlosser, 1989; Sheldon, 1968), higher food web productivity and stability, and enhanced protection from physical disturbances (Bremigan et al., 2008; Graham & Nash, 2013). Similarly, the environmental variables considered in this study, such as land cover patch composition and vegetation height variation, were correlated with habitat heterogeneity and were distributed as mid‐peaked patterns along the analyzed submontane rivers (Figure S1), which may lead to the spatial variations in the multiple diversity of birds in this study.

### 4.2 Environmental drivers of the biodiversity pattern of birds

The natural longitudinal changes in the biodiversity of birds are attributed to both riverine terrestrial attributes and hydrogeomorphologies along submontane rivers. Bird taxonomic diversity indices (species richness and Shannon diversity) were positively related to forest cover, vegetation height variation, and land cover patches. The available forest areas along bodies of water, which serve as habitats and important ecological corridors, support some of the most individual‐ and species‐rich avian communities, although riparian forests are often just a small part of the landscape (Gomez et al., 2021; Groom & Grubb, 2004; Hsu et al., 2010). Continuous and large areas of riparian forests could meet the requirements of various bird species with regard to habitat size and certain special resources, such as nest holes for birds that inhabit forest or river habitats, thus allowing abundant bird species to coexist (Lees & Peres, 2007; Magdaleno & Martinez, 2014). Moreover, increasing forest areas have been found to allow species of various body sizes to forage, thus increasing the local natural disturbance level and resulting in greater microhabitat availability related to habitat complexity (Kovalenko et al., 2012). In accordance with other studies (Jankowski et al., 2013; Zhang et al., 2013), the relatively high vegetation height variability (reflecting the vertical heterogeneity of riparian forest) could also contribute to the increased bird taxonomic diversity in this case, which may reflect higher niche segregation opportunities, as it was linked to stratification of the forest, resulting in different layers (different niches) among the ground‐, shrub‐ and canopy‐breeding/foraging birds (Parker & Brown, 2000). Forest cover may fulfill needs based on the amount of resources, whereas vertical vegetation heterogeneity may fulfill needs based on how varied the spatial arrangements of these resources are. For example, some species of passerine require shrub cover to nest and refuge against predators (Perkins et al., 2000), but they also require access to varying amounts of both vegetation structure and open ground within a site for foraging (Benton et al., 2003; Gorini et al., 2012). In addition, landscapes containing diverse land cover types further improved the taxonomic diversity of riparian birds. According to the habitat heterogeneity hypothesis (MacArthur & MacArthur, 1961), resources and niches increase with increasing spatial heterogeneity (Bazzaz, 1975; Pianka, 1972). More land cover patches allowed more species coexistence by fulfilling multiple bird guilds resource and habitat needs and by weakening competitive interactions (Fahrig et al., 2011; Rosenzweig, 1995; Warfe & Barmuta, 2006).

Naturally, meandering rivers (with asymmetrical bathymetries and diverse flow patterns) exhibit cross‐sectional diversity in their physical properties, which are more complex than the properties of straight streams and thus promote an increase in species diversity (Nakano & Nakamura, 2008). However, river sinuosity was found in this study to be positively related to species richness but not to Shannon diversity. The meandering river reaches easily form pool‐riffle sequences at river bends (Beschta & Platts, 1986), and the slow velocity and abundant resources in pool‐riffle sequences allow river‐dwelling birds, especially piscivorous birds to forage. These habitat specialists, however, are generally less common than forest‐dwelling birds such as passerines, which may result in an uneven community distribution and, in turn, nonsignificant relationships with Shannon‐Winner diversity.

During this study, phylogenetic diversity was found to be significantly related to fluvial geomorphology variables other than terrestrial attributes. Previous studies proposed that PD was increased with species amounts and relatedness (Frishkoff et al., 2014). Increased river sinuosity can provide high‐quality habitats for both river‐dwelling and forest‐dwelling species due to the increased amount of space close to the river edge and area (Bertalan et al., 2018). River reaches with higher sinuosity levels may thus support assemblages with diverse phylogenetic relationships, as river‐dwelling species usually have further phylogenetic distances from forest‐dwelling species (Figure S2). In contrast to the relationship to sinuosity, phylogenetic diversity was negatively related to gravel bars. The abundance and richness of aquatic insects (important energy sources for aquatic and terrestrial flycatchers and gleaners) in riparian zones decreased with the distance between the water body and forest edge (Iwata et al., 2003). Gravel bars along the river edge increase the distance between water bodies and the forest edge and may therefore reduce the food availability and lead to a decline in insect‐eating bird guilds, in turn reducing the phylogenetic diversity.

Functional diversity metrics can reflect the amount of inter‐specific variation in functional traits of a group of species and provide an index of niche complementarity and the diversity of ecological interactions (Petchey & Gaston, 2006). However, we did not find any significant correlations between FD and environmental variables, although the mid‐peak pattern was apparent. We believe that the two processes may be the causative mechanisms explaining the nonsignificant response of the FD indices in this study. First, FD measurements require a higher sample size than taxonomic diversity measurements to ensure the accuracy of the results (van der Plas et al., 2017). The sampling data we collected could be insufficient for capturing FD. Second, FD and PD are considered to be related to the spatial scale of sampling (Calba et al., 2014). In our survey, the distance between some adjacent sampling sites was less than 2 km, which may have led to the high sensitivity of FD (Calba et al., 2014).

Although the multiple dimensions of bird community diversity showed similar distribution patterns along the analyzed river valleys, the responses of taxonomic diversity, functional diversity, and phylogenetic diversity to riparian landscape configuration were inconsistent, which provided additional information for biodiversity and indicated that studying different aspects of the diversity of species assemblages can help researchers better understand the mechanisms that generate and maintain diversity patterns. To our knowledge, this is one of the few studies to document longitudinal distribution patterns of multiple dimensions of diversity in bird communities along submontane rivers (Sinha et al., 2022). This study expands our general understanding of bird distribution patterns along longitudinal gradients and their driving factors in submontane riparian systems. The findings emphasize the necessity of including multiple diversity measures beyond taxonomic diversity and demonstrate the importance of both terrestrial and aquatic components in shaping the multifaceted biodiversity pattern of riparian birds and likely other organisms, such as mustelids and frogs, living among aquatic‐terrestrial ecotones.

## 5 CONSERVATION IMPLICATIONS

**FIGURE 4:**
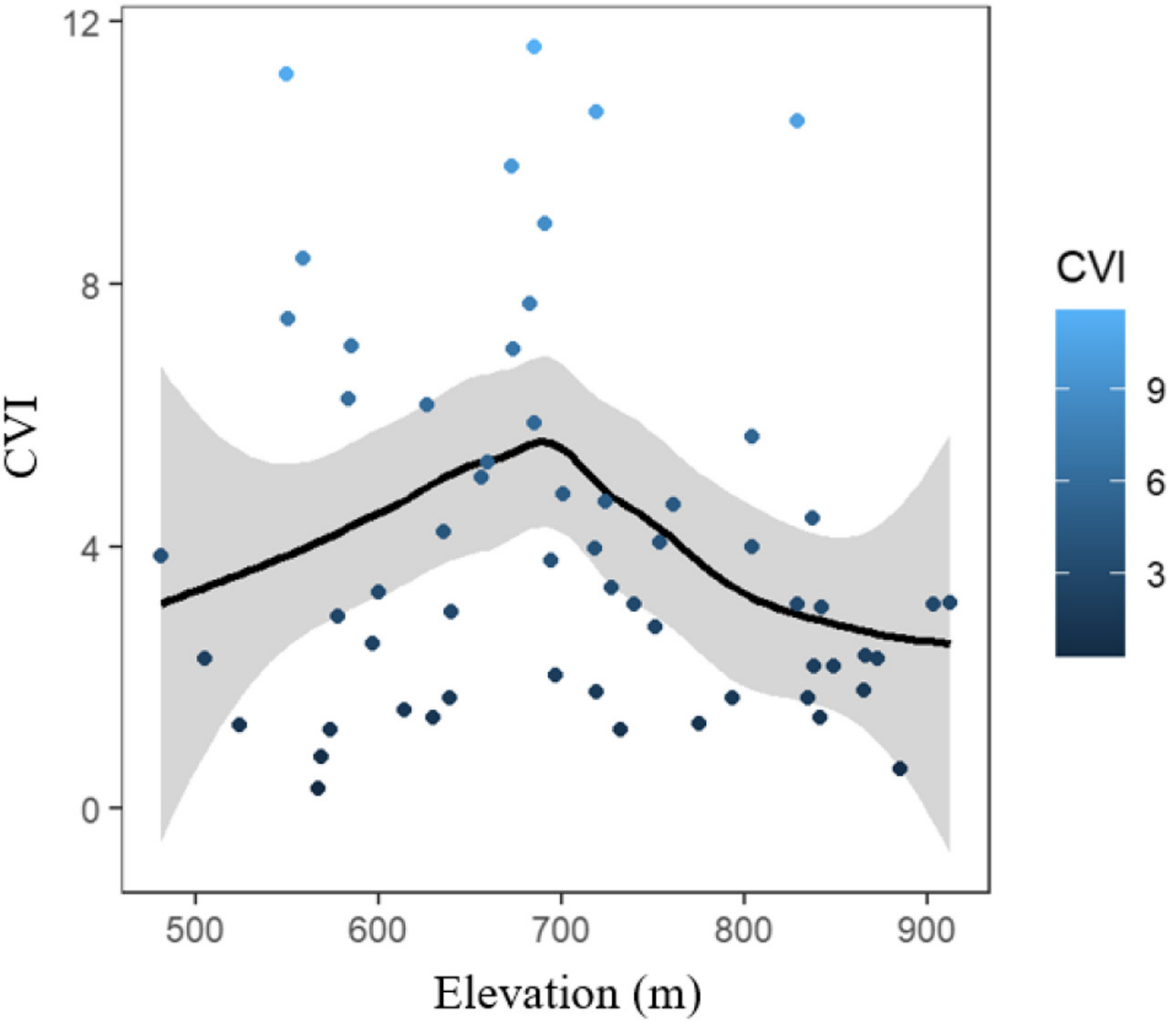
Relationship between conservation value index (CVI) and elevations. Solid lines represent trends, and the gray area represents the 95% confidence intervals fitted by generalized linear models.

Mountainous riparian ecosystems provide numerous services and are of critical conservation concern under human disturbances (Patten, 1998; Zaimes et al., 2021). However, our knowledge of anthropogenic threats to the biodiversity and environments of this ecotone remains limited due to its sparse distribution, relatively small extent, and limited accessibility (Kajtoch et al., 2016). The mid‐peaked distribution of multiple dimensions of diversity and the higher CVI value (containing more threatened species; Figure 4) of bird communities in the mid‐reaches of the analyzed rivers emphasize the conservation priority for middle reaches to maintain biodiversity and ecosystem services. The significant correlations between riparian birds and aquatic/terrestrial habitat elements (thereby possessing important ecological values in supporting higher taxonomic and phylogenetic bird diversity) suggest that riparian bird communities could be used as good candidate indicators of environmental changes in the submontane stream forest of the Changbai Mountains and probably in other mountainous areas.

## AUTHOR CONTRIBUTIONS

**Wenyu Xu:** Data curation (equal); formal analysis (lead); investigation (lead); methodology (lead); resources (equal); software (equal); visualization (lead); writing – original draft (lead). **Ye Gong:** Conceptualization (lead); funding acquisition (equal); project administration (equal); supervision (equal); validation (lead); writing – review and editing (lead). **Lin Wang:** Resources (equal); software (equal); validation (equal). **Jiyuan Yao:** Investigation (equal). **Haitao Wang:** Funding acquisition (lead); project administration (lead); supervision (equal); writing – review and editing (equal).

## CONFLICT OF INTEREST

The authors declare no conflicts of interest.

## Supporting information


Appendix S1

## Data Availability

The datasets analyzed during the current study are available in the Zenodo repository at https://doi.org/10.5281/zenodo.7016118.

## References

[ece39542-bib-0001] Abdelsalam, K. M. (2012). Benthic macro‐ and meso‐invertebrates of a sandy riverbed in a mountain stream, Central Japan. Limnology, 13(1), 171–179.

[ece39542-bib-0002] Angermeier, P. L. , & Schlosser, I. J. (1989). Species‐area relationship for stream fishes. Ecology, 70(5), 1450–1462.

[ece39542-bib-0003] Bae, S. , Muller, J. , Lee, D. , Vierling, K. T. , Vogeler, J. C. , Vierling, L. A. , Hudak, A. T. , Latifi, H. , & Thorn, S. (2018). Taxonomic, functional, and phylogenetic diversity of bird assemblages are oppositely associated to productivity and heterogeneity in temperate forests. Remote Sensing of Environment, 215, 145–156.

[ece39542-bib-0004] Bates, D. , Maechler, M. , Bolker, B. , & Walker, S. (2014). lme4: Linear mixed‐effects models using Eigen and S4, R package version 1.1‐7. https://CRAN.R‐project.org/package=lme4

[ece39542-bib-0005] Bazzaz, F. A. (1975). Plant species diversity in old‐field successional ecosystems in southern Illinois. Ecology, 56(2), 485–488.

[ece39542-bib-0006] Begley, M. T. , & Krebs, R. A. (2017). Application of OEPA‐produced biotic indices and physical stream measurements to assess freshwater mussel (Unionidae) habitat in the upper Mahoning River, Ohio. Northeastern Naturalist, 24(1), 1–14.

[ece39542-bib-0007] Benton, T. G. , Vickery, J. A. , & Wilson, J. D. (2003). Farmland biodiversity: Is habitat heterogeneity the key? Trends in Ecology & Evolution, 18, 182–188.

[ece39542-bib-0008] Berrahou, A. , Cellot, B. , & Richoux, R. (2001). Longitudinal distribution of benthic macroinvertebrates in the Moulouya River (Morocco). Annales de Limnologie‐International Journal of Limnology, 37(3), 223–235.

[ece39542-bib-0009] Bertalan, L. , Novak, T. J. , Nemeth, Z. , Rodrigo‐Comino, J. , Kertesz, A. , & Szabo, S. (2018). Issues of meander development: Land degradation or ecological value? The example of the Sajo River, Hungary. Water, 10(11), 1613.

[ece39542-bib-0010] Beschta, R. L. , & Platts, W. S. (1986). Morphological features of small streams: Significance and function. Journal of the American Water Works Association, 22, 369–379.

[ece39542-bib-0011] Bibby, C. J. , Burgess, N. D. , Hill, D. A. , & Mustoe, S. (2000). Bird census techniques. Academic.

[ece39542-bib-0012] Blomberg, S. P. , Garland, T. , & Ives, A. R. (2003). Testing for phylogenetic signal in comparative data: Behavioral traits are more labile. Evolution, 57, 717–745. 12778543 10.1111/j.0014-3820.2003.tb00285.x

[ece39542-bib-0013] Bremigan, M. T. , Soranno, P. A. , Gonzalez, M. J. , Bunnell, D. B. , Arend, K. K. , Renwick, W. H. , Stein, R. A. , & Vanni, M. J. (2008). Hydrogeomorphic features mediate the effects of land use/cover on reservoir productivity and food webs. Limnology and Oceanography, 53(4), 1420–1433.

[ece39542-bib-0014] Burnham, K. P. , & Anderson, D. R. (2002). Model selection and multimodel inference: A practical information‐theoretic approach. Springer‐Verlag.

[ece39542-bib-0015] Calba, S. , Maris, V. , & Devictor, V. (2014). Measuring and explaining large‐scale distribution of functional and phylogenetic diversity in birds: Separating ecological drivers from methodological choices. Global Ecology and Biogeography, 23(6), 669–678.

[ece39542-bib-0016] Canterbury, G. E. , Martin, T. E. , Petit, D. R. , Petit, L. J. , & Bradford, D. F. (2000). Bird communities and habitat as ecological indicators of forest condition in regional monitoring. Conservation Biology, 14(2), 544–558.

[ece39542-bib-0017] Carvalho‐Rocha, V. , Peres, C. A. , & Neckel‐Oliveira, S. (2020). Habitat amount and ambient temperature dictate patterns of anuran diversity along a subtropical elevational gradient. Diversity and Distribution, 27(2), 344–359.

[ece39542-bib-0018] Corenblit, D. , Davies, N. S. , Steiger, J. , Gibling, M. R. , & Bornette, G. (2014). Considering river structure and stability in the light of evolution: Feedbacks between riparian vegetation and hydrogeomorphology. Earth Surface Process and Landforms, 40(2), 189–207.

[ece39542-bib-0019] Cornwell, W. K. , Schwilk, D. W. , & Ackerly, D. D. (2006). A trait–based test for habitat filtering: Convex hull volume. Ecology, 87, 1465–1471. 16869422 10.1890/0012-9658(2006)87[1465:attfhf]2.0.co;2

[ece39542-bib-0020] Cubley, E. S. , Bateman, H. L. , Merritt, D. M. , & Cooper, D. J. (2020). Using vegetation guilds to predict bird habitat characteristics in riparian areas. Wetlands, 40(6), 1843–1862.

[ece39542-bib-0021] Ding, Z. F. , Hu, H. J. , Cadotte, M. W. , Liang, J. C. , Hu, Y. M. , & Si, X. F. (2021). Elevational patterns of bird functional and phylogenetic structure in the central Himalaya. Ecography, 44(9), 1403–1417.

[ece39542-bib-0022] ESRI . (2014). ArcGIS desktop: Release 10.2. Environmental Systems Research Institute.

[ece39542-bib-0023] Fahrig, L. , Baudry, J. , Brotons, L. , Burel, F. G. , Crist, T. O. , Fuller, R. J. , Sirami, C. , Siriwardena, C. M. , & Martin, J. L. (2011). Functional landscape heterogeneity and animal biodiversity in agricultural landscapes. Ecology Letters, 14, 101–112. 21087380 10.1111/j.1461-0248.2010.01559.x

[ece39542-bib-0024] Finn, D. S. , & Poff, N. L. (2005). Variability and convergence in benthic communities along the longitudinal gradients of four physically similar Rocky Mountain streams. Freshwater Biology, 50(2), 243–261.

[ece39542-bib-0025] Fleishman, E. , McDonald, N. , MacNally, R. , Murphy, D. D. , Walters, J. , & Floyd, T. (2003). Effects of floristics, physiognomy and non‐native vegetation on riparian bird communities in a Mohave Desert watershed. Journal of Animal Ecology, 72, 484–490.

[ece39542-bib-0026] Foster, W. A. , Snaddon, J. L. , Turner, E. C. , Fayle, T. M. , Cockerill, T. D. , Ellwood, M. D. F. , Broad, G. R. , Chung, A. Y. C. , Eggleton, P. , Khen, C. V. , & Yusah, K. M. (2011). Establishing the evidence base for maintaining biodiversity and ecosystem function in the oil palm landscapes of South East Asia. Philosophical Transactions of the Royal Society B‐Biological Sciences, 366(1582), 3277–3291. 10.1098/rstb.2011.0041 PMC3179631 22006968

[ece39542-bib-0027] Frishkoff, L. O. , Karp, D. S. , M'Gonigle, L. K. , Mendenhall, C. D. , Zook, J. , Kremen, C. , Hadly, E. A. , & Daily, G. C. (2014). Loss of avian phylogenetic diversity in neotropical agricultural systems. Science, 345, 1343–1346. 25214627 10.1126/science.1254610

[ece39542-bib-0028] Gomez, D. , Ruggera, R. A. , Rivera, L. O. , & Politi, N. (2021). Patterns of taxonomic and functional diversity of birds in riparian forests with natural and agricultural matrices in the argentine austral Yungas. Ibis, 163(3), 845–857.

[ece39542-bib-0029] Gorini, L. , Linnell, J. D. C. , May, R. , Panzacchi, M. , Boitani, L. , Odden, M. , & Nilsen, E. B. (2012). Habitat heterogeneity and mammalian predator–prey interactions. Mammal Review, 42, 55–77.

[ece39542-bib-0030] Graham, N. A. J. , & Nash, K. L. (2013). The importance of structural complexity in coral reef ecosystems. Coral Rees, 32(2), 315–326.

[ece39542-bib-0031] Groom, J. D. , & Grubb, T. C. (2004). Bird species associated with riparian woodland in fragmented, temperate‐deciduous forest. Conservation Biology, 16(3), 832–836.

[ece39542-bib-0032] Han, Z. , Zhang, L. S. , Jiang, Y. L. , Wang, H. T. , & Jiguet, F. (2020). Unravelling species co‐occurrence in a steppe bird community of Inner Mongolia: Insights for the conservation of the endangered Jankowski's bunting. Diversity and Distribution, 26(7), 843–852.

[ece39542-bib-0033] Hanle, J. , Duguid, M. C. , & Ashton, M. S. (2020). Legacy forest structure increases bird diversity and abundance in aging young forests. Ecology and Evolution, 10(3), 1193–1208. 32076507 10.1002/ece3.5967 PMC7029076

[ece39542-bib-0034] He, X. L. , Luo, K. , Brown, B. , & Lin, L. X. (2018). A taxonomic, functional, and phylogenetic perspective on the community assembly of passerine birds along an elevational gradient in Southwest China. Ecology and Evolution, 8(5), 2712–2720. 29531688 10.1002/ece3.3910 PMC5838049

[ece39542-bib-0035] Hijmans, R. , & Van Etten, J. (2012). Raster: Geographic data analysis and modelling. R package version.

[ece39542-bib-0036] Hill, M. O. (1973). Diversity and evenness: A unifying notation and its consequences. Ecology, 54, 427–431.

[ece39542-bib-0037] Hsu, T. N. , French, K. , & Major, R. (2010). Avian assemblages in eucalypt forests, plantations and pastures in northern NSW, Australia. Forest Ecology and Management, 260(6), 1036–1046.

[ece39542-bib-0038] Hurlbert, A. H. (2004). Species‐energy relationships and habitat complexity in bird communities. Ecology Letters, 7(8), 714–720.

[ece39542-bib-0039] Iwata, T. , Nakano, S. , & Murakami, M. (2003). Stream meanders increase insectivorous bird abundance in riparian deciduous forests. Ecography, 26(3), 325–337.

[ece39542-bib-0040] Jankowski, J. E. , Merkord, C. L. , Rios, W. F. , Cabrera, K. G. , Revilla, N. S. , & Silman, M. R. (2013). The relationship of tropical bird communities to tree species composition and vegetation structure along an Andean elevational gradient. Journal of Biogeography, 40, 950–962.

[ece39542-bib-0041] Kajtoch, L. , Wilk, T. , Bobrek, R. , & Matysek, M. (2016). The importance of forests along submontane stream valleys for bird conservation: The Carpathian example. Bird Conservation International, 26, 350–365.

[ece39542-bib-0042] Kembel, S. W. , Cowan, P. D. , Helmus, M. R. , Cornwell, W. K. , Morlon, H. , Ackerly, D. D. , Blomberg, S. P. , & Webb, C. O. (2010). Picante: R tools for integrating phylogenies and ecology. Bioinformatics, 26, 1463–1464. 20395285 10.1093/bioinformatics/btq166

[ece39542-bib-0043] Kovalenko, K. E. , Thomaz, S. M. , & Warfe, D. M. (2012). Habitat complexity: Approaches and future directions. Hydrobiologia, 685(1), 1–17.

[ece39542-bib-0044] Laliberte, E. , & Legendre, P. (2010). A distance‐based framework for measuring functional diversity from multiple traits. Ecology, 91(1), 299–305. 20380219 10.1890/08-2244.1

[ece39542-bib-0045] Lees, A. C. , & Peres, C. A. (2007). Conservation value of remnant riparian forest corridors of varying quality for Amazonian birds and mammals. Conservation Biology, 22(2), 439–449. 10.1111/j.1523-1739.2007.00870.x 18241239

[ece39542-bib-0046] Li, Y. , Chang, Y. , He, X. Y. , Xu, S. , & Su, D. Y. (2022). Effect of environmental factors on the spatial diversity distribution patterns of lycophytes and ferns in Northeast China. Russian Journal of Ecology, 53(2), 111–122.

[ece39542-bib-0047] Li, Y. R. , Tao, J. , Chu, L. , & Yan, Y. Z. (2018). Effects of anthropogenic disturbances on α and β diversity of fish assemblages and their longitudinal patterns in subtropical streams, China. Ecology of Freshwater Fish, 27, 433–441.

[ece39542-bib-0048] Losos, J. B. (2008). Phylogenetic niche conservatism, phylogenetic signal and the relationship between phylogenetic relatedness and ecological similarity among species. Ecology Letters, 11, 995–1003. 18673385 10.1111/j.1461-0248.2008.01229.x

[ece39542-bib-0049] Lukacs, P. M. , Burnham, K. P. , & Anderson, D. R. (2009). Model selection bias and Freedman's paradox. Annals of the Institute of Statistical Mathematics, 62(1), 117–125.

[ece39542-bib-0050] MacArthur, R. H. , & MacArthur, J. W. (1961). On bird species diversity. Ecology, 42, 594–598.

[ece39542-bib-0051] Magdaleno, F. , & Martinez, R. (2014). Evaluating the quality of riparian forest vegetation: The riparian Forest evaluation (RFV) index. Forest Systems, 23(2), 259–272.

[ece39542-bib-0052] McCain, C. M. (2007). Could temperature and water availability drive elevational species richness patterns? A global case study forbats. Global Ecology and Biogeography, 16, 1–13.

[ece39542-bib-0053] Naiman, R. J. , Decamps, H. , & Pollock, M. (1993). The role of riparian corridors in maintaining regional biodiversity. Ecological Applications, 3(2), 209–212. 27759328 10.2307/1941822

[ece39542-bib-0054] Nakano, D. , & Nakamura, F. (2008). The significance of meandering channel morphology on the diversity and abundance of macroinvertebrates in a lowland river in Japan. Aquatic Conservation‐Marine and Freshwater Ecosystems, 18(5), 780–798.

[ece39542-bib-0055] Palmer, G. C. , & Bennett, A. F. (2006). Riparian zones provide for distinct bird assemblages in forest mosaics of south‐East Australia. Biological Conservation, 130(3), 447–457.

[ece39542-bib-0056] Parker, G. G. , & Brown, M. J. (2000). Forest canopy stratification‐is it useful? American Naturalist, 155, 473–484. 10.1086/303340 10753075

[ece39542-bib-0057] Patten, D. T. (1998). Riparian ecosystems of semi‐arid North America: Diversity and human impacts. Wetlands, 18(4), 498–512.

[ece39542-bib-0058] Perkins, A. J. , Whittingham, M. J. , Bradbury, R. B. , Wilson, J. D. , Morris, A. J. , & Barnett, P. R. (2000). Habitat characteristics affecting use of lowland agricultural grassland by birds in winter. Biological Conservation, 95, 279–294.

[ece39542-bib-0059] Petchey, O. L. (2004). On the statistical significance of functional diversity. Functional Ecology, 18(3), 297–303.

[ece39542-bib-0060] Petchey, O. L. , & Gaston, K. J. (2006). Functional diversity: Back to basics and looking forward. Ecology Letters, 9(6), 741–758. 16706917 10.1111/j.1461-0248.2006.00924.x

[ece39542-bib-0061] Pianka, E. R. (2011). Evolutionary ecology. Pianka Eric R.

[ece39542-bib-0062] Ping, Y. A. , Han, D. X. , Wang, N. , Hu, Y. B. , Mu, L. Q. , & Feng, F. J. (2017). Vertical zonation of soil fungal community structure in a Korean pine forest on Changbai Mountain, China. World Journal of Microbiology & Biotechnology, 33(1), 12. 27885566 10.1007/s11274-016-2133-1

[ece39542-bib-0063] Pons, P. , Lambert, B. , Rigolot, E. , & Prodon, R. (2003). The effects of grassland management using fire on habitat occupancy and conservation of birds in a mosaic landscape. Biodiversity and Conservation, 12(9), 1843–1860.

[ece39542-bib-0064] Qi, L. , Zhao, F. Q. , & Sun, J. (2018). An integrated multi‐scale approach to restoring a degraded secondary forest ecosystem: A case study in the Changbai Mountains, northeastern China. Ecological Engineering, 125, 98–105.

[ece39542-bib-0065] R Core Team . (2014). R: A language and environment for statistical computing. R Foundation for statistical Computing. http://www.R‐project.org/

[ece39542-bib-0066] Rahbek, C. (1997). The relationship among area, elevation, and regional species richness in neotropical birds. The American Naturalist, 149, 875–902. 10.1086/286028 18811253

[ece39542-bib-0067] Rakotomalala, Z. , & Goodman, S. M. (2010). Diversity and longitudinal species turnover of small mammals in the forests of watersheds of western Madagascar. Revue D Ecologie, 65(4), 343–358.

[ece39542-bib-0068] Randall, R. G. , Minns, C. K. , Cairns, V. W. , & Moore, J. E. (1996). The relationship between an index of fish production and submerged macrophytes and other habitat features at three littoral areas in the Great Lakes. Canadian Journal of Fisheries and Aquatic Sciences, 53(S1), 35–44.

[ece39542-bib-0069] Resh, V. H. , Brown, A. V. , Covich, A. P. , Gurtz, M. E. , Li, H. W. , Minshall, G. W. , Reice, S. R. , Sheldon, A. L. , & Wissmar, R. C. (1988). The role of disturbance in stream ecology. Journal of the North American Benthological Society, 7, 433–455.

[ece39542-bib-0070] Rosenzweig, M. L. (1995). Species diversity in space and time. Cambridge University Press.

[ece39542-bib-0071] Royan, A. , Hannah, D. M. , Reynold, S. J. , Noble, D. G. , & Sadler, J. P. (2014). River birds' response to hydrological extremes: New vulnerability index and conservation implications. Biological Conservation, 177, 64–73.

[ece39542-bib-0072] Royan, A. , Reynolds, S. J. , Hannah, D. M. , Prudhomme, C. , Noble, D. G. , & Sadler, J. P. (2015). Shared environmental responses drive co‐occurrence patterns in river bird communities. Ecography, 39(8), 733–742.

[ece39542-bib-0073] Sheldon, A. L. (1968). Species diversity and longitudinal succession in stream fishes. Ecology, 49(2), 194–198.

[ece39542-bib-0074] Sinha, A. , Chatterjee, N. , Krishnamurthy, R. , & Ormerod, S. J. (2022). Community assembly, functional traits, and phylogeny in Himalayan river birds. Ecology and Evolution, 12(6), e9012. 35784086 10.1002/ece3.9012 PMC9204853

[ece39542-bib-0075] Smiley, P. C. , Knight, S. S. , Shields, F. D. , & Cooper, C. M. (2009). Influence of gully erosion control on amphibian and reptile communities within riparian zones of channelized streams. Ecohydrology, 2(3), 303–312.

[ece39542-bib-0076] Steward, A. L. , Datry, T. , & Langhans, S. D. (2022). The terrestrial and semi‐aquatic invertebrates of intermittent rivers and ephemeral streams. Biological Reviews, 97(4), 1408–1425. 35229438 10.1111/brv.12848 PMC9542210

[ece39542-bib-0077] Swenson, N. G. (2011). The role of evolutionary processes in producing biodiversity patterns, and the interrelationships between taxonomic, functional and phylogenetic biodiversity. American Journal of Botany, 98, 472–480. 21613140 10.3732/ajb.1000289

[ece39542-bib-0078] Terraube, J. , Archaux, F. , Deconchat, M. , van Halder, I. , Jactel, H. , & Barbaro, L. (2016). Forest edges have high conservation value for bird communities in mosaic landscapes. Ecology and Evolution, 6, 5178–5189. 27551375 10.1002/ece3.2273 PMC4984496

[ece39542-bib-0079] Torgersen, C. E. , Baxter, C. V. , Li, H. W. , & McIntosh, B. A. (2006). Landscape influences on longitudinal patterns of river fishes: Spatially continuous analysis of fish‐habitat relationships. In American Fisheries Society Symposium, Vol. 48 (p. 473). American Fisheries Society.

[ece39542-bib-0080] Usseglio‐Polatera, P. , & Beisel, J. N. (2002). Longitudinal changes in macroinvertebrate assemblages in the Meuse River: Anthropogenic effects versus natural change. River Research and Applications, 18(2), 197–211.

[ece39542-bib-0081] van der Plas, F. , van Klink, R. , Manning, P. , Olff, H. , & Fischer, M. (2017). Sensitivity of functional diversity metrics to sampling intensity. Methods in Ecology and Evolution, 8(9), 1072–1080.

[ece39542-bib-0082] Vannote, R. L. , Minshall, G. W. , Cummins, K. W. , Sedell, J. R. , & Cushing, C. E. (1980). The river continuum concept. Canadian Journal of Fisheries and Aquatic Sciences, 37, 130–137.

[ece39542-bib-0083] Wang, X. D. , Li, S. , Price, M. , Lei, Y. , Wu, B. , Liu, K. , & Song, Z. B. (2019). Longitudinal and seasonal patterns of fish assemblage structure in the Zhougong River, Sichuan Province, Southwest China. Ecological Indicators, 107, 105656.

[ece39542-bib-0084] Ward, J. V. (1989). The four‐dimensional nature of lotic ecosystems. Journal of the North American Benthological, 8, 2–8.

[ece39542-bib-0085] Ward, J. V. (1998). Riverine landscapes: Biodiversity patterns, disturbance regimes, and aquatic conservation. Biological Conservation, 83(3), 269–278.

[ece39542-bib-0086] Warfe, D. M. , & Barmuta, L. A. (2006). Habitat structural complexity mediates food web dynamics in a freshwater macrophyte community. Oecologia, 150(1), 141–154. 16932971 10.1007/s00442-006-0505-1

[ece39542-bib-0087] Wood, S. N. (2011). Fast stable restricted maximum likelihood and marginal likelihood estimation of semiparametric generalized linear models. Journal of the Royal Statistical Society Series B‐Statistical Methodology, 73, 3–36.

[ece39542-bib-0088] Wu, W. , Xu, H. , Wu, J. , & Cao, M. (2012). The impact of climate change on birds: A review. Biodiversity Science, 20(1), 108–115.

[ece39542-bib-0089] Xu, W. Y. , Wang, L. , Gong, Y. , & Wang, H. T. (2021). The indicator roles of endangered scaly sided merganser (Mergus squamatus) in submontane rivers of Changbai Mountains, China. Ecological Indicators, 129, 107966.

[ece39542-bib-0090] Zaimes, G. N. , Iakovoglou, V. , Syropoulos, D. , Kaltsas, D. , & Avtzis, D. (2021). Assessment of two adjacent mountainous riparian areas along Nestos River tributaries of Greece. Forests, 12(9), 1284.

[ece39542-bib-0091] Zhang, D. , Heng, W. J. , Chu, L. , Xu, D. P. , Kang, B. , & Yan, Y. Z. (2020). Taxonomic and functional diversity in a subtropical stream: A longitudinal pattern analysis. Ecology of Freshwater Fish, 29(4), 752–763.

[ece39542-bib-0092] Zhang, J. , Kissling, W. D. , & He, F. (2013). Local forest structure, climate and human disturbance determine regional distribution of boreal bird species richness in Alberta, Canada. Journal of Biogeography, 40, 1131–1142.

