# Supplementary material for: Longitudinal multidiversity pattern and the environmental drivers of riparian bird communities along submontane rivers of Changbai Mountains, China
Source: Ecol Evol. 2022 Nov 27;12(11):e9542. doi: 10.1002/ece3.9542 (PMC9702571; doi:10.1002/ece3.9542)
Supplement: Supplementary file 1 — Appendix S1 [file ECE3-12-e9542-s001.docx]

**Table S1. Land cover types derived from the satellite imagery.**

| **Code** | **Class Name** | **Code** | **Class Name** |
| --- | --- | --- | --- |
| 1 | Broadleaf Evergreen Forest | 11 | Cropland |
| 2 | Broadleaf Deciduous Forest | 12 | Paddy field |
| 3 | Needleleaf Evergreen Forest | 13 | Cropland/Other Vegetation Mosaic |
| 4 | Needleleaf Deciduous Forest | 14 | Mangrove |
| 5 | Mixed Forest | 15 | Wetland |
| 6 | Tree Open | 16 | Bare area, consolidated (grave, rock) |
| 7 | Shrub | 17 | Bare area, unconsolidated (sand) |
| 8 | Herbaceous | 18 | Urban |
| 9 | Herbaceous with Sparse Tree/Shrub | 19 | Snow/Ice |
| 10 | Sparse vegetation | 20 | Water bodies |

**Table S2. Functional traits of birds used in this study. Habitat specialization was counted as the number of habitat types that species is known to occupy, lower value indicates higher habitat specificity.** **Clutch size was defined as the median number of eggs per nest. Abundance is the total number of observed individuals**

**across all sampling sites. *represents the species included in the analysis.**

| Scientific name | Habitat specifity | Nest site | Migrant status | Trophic level | Body mass | Clutch size | Abundance |
| --- | --- | --- | --- | --- | --- | --- | --- |
| Hirundo_rustica* | 6 | Rock | Full migrant | Insectivores | 20 | 4.5 | 79 |
| Emberiza_spodocephala* | 3 | Shrub | Full migrant | Omnivores | 19 | 5 | 71 |
| Phylloscopus_proregulus* | 1 | Cavity | Full migrant | Insectivores | 6 | 4.5 | 55 |
| Aix_galericulata* | 2 | Cavity | Full migrant | Piscivores | 501 | 10 | 50 |
| Passer_montanus* | 3 | Cavity | Resident | Omnivores | 24 | 4.5 | 50 |
| Motacilla_cinerea* | 4 | Water | Full migrant | Insectivores | 17.5 | 5 | 45 |
| Anas platyrhynchos* | 2 | Ground | Full migrant | Herbivores | 1082 | 9 | 41 |
| Actitis_hypoleucos* | 7 | Ground | Full migrant | Insectivores | 50 | 4.5 | 40 |
| Turdus_hortulorum* | 2 | Shrub | Full migrant | Omnivores | 242.5 | 2 | 29 |
| Phylloscopus_coronatus* | 2 | Ground | Full migrant | Insectivores | 8.5 | 5.5 | 28 |
| Cyanoptila_cyanomelana* | 3 | Rock | Full migrant | Insectivores | 23 | 4 | 28 |
| Urosphena_squameiceps* | 4 | Ground | Full migrant | Insectivores | 9 | 5.5 | 26 |
| Phoenicurus_auroreus* | 3 | Cavity | Full migrant | Insectivores | 17 | 6.5 | 25 |
| Mergus_squamatus* | 3 | Cavity | Full migrant | Piscivores | 999 | 10 | 22 |
| Parus_major* | 5 | Cavity | Resident | Omnivores | 14 | 7.5 | 20 |
| Emberiza_elegans* | 3 | Shrub | Resident | Insectivores | 17 | 6 | 19 |
| Motacilla_alba* | 5 | Rock | Full migrant | Insectivores | 24 | 5.5 | 18 |
| Streptopelia_orientalis* | 4 | Tree | Partial migrant | Herbivores | 219.5 | 2 | 18 |
| Cecropis_daurica* | 5 | Rock | Full migrant | Insectivores | 23.5 | 5 | 16 |
| Phasianus_colchicus* | 3 | Ground | Resident | Omnivores | 1263 | 11.5 | 16 |
| Carpodacus_sibiricus* | 3 | Shrub | Full migrant | Omnivores | 19 | 5 | 14 |
| Phylloscopus inornatus* | 3 | Ground | Full migrant | Insectivores | 7.5 | 3.5 | 12 |
| Cuculus_saturatus* | 2 | Tree | Full migrant | Insectivores | 104 | 10 | 11 |
| Cyanopica_cyanus* | 2 | Tree | Full migrant | Omnivores | 95 | 7 | 11 |
| Corvus_corone* | 8 | Tree | Full migrant | Omnivores | 375 | 2.5 | 9 |
| Phylloscopus_trochiloides* | 4 | Ground | Resident | Insectivores | 8.5 | 5.5 | 8 |
| Turdus_pallidus* | 3 | Shrub | Resident | Omnivores | 72 | 5 | 7 |
| Lanius_cristatus* | 5 | Shrub | Full migrant | Insectivores | 31.5 | 5 | 7 |
| Parus_montanus* | 4 | Cavity | Full migrant | Omnivores | 11 | 8 | 7 |
| Pericrocotus_divaricatus* | 3 | Tree | Full migrant | Insectivores | 24 | 4.5 | 6 |
| Sitta_europaea* | 1 | Cavity | Resident | Omnivores | 18 | 7 | 6 |
| Ficedula_zanthopygia* | 3 | Cavity | Full migrant | Omnivores | 12 | 5.5 | 6 |
| Coccothraustes_coccothraustes* | 2 | Tree | Resident | Omnivores | 53 | 4.5 | 6 |
| Garrulus glandarius* | 2 | Tree | Full migrant | Omnivores | 155 | 6.5 | 4 |
| Anas_poecilorhyncha* | 4 | Ground | Full migrant | Omnivores | 1102.5 | 10 | 4 |
| Alcedo_atthis* | 7 | Cavity | Resident | Piscivores | 29 | 6 | 4 |
| Cuculus_micropterus* | 2 | Tree | Full migrant | Insectivores | 119 | 10 | 4 |
| Muscicapa_dauurica* | 2 | Tree | Full migrant | Omnivores | 11 | 5 | 4 |
| Picus_canus* | 2 | Cavity | Resident | Insectivores | 133.5 | 9.5 | 4 |
| Carduelis_sinica* | 4 | Tree | Full migrant | Herbivores | 18 | 4 | 3 |
| Parus_palustris* | 4 | Cavity | Resident | Omnivores | 12 | 8 | 3 |
| Dendrocopos_major* | 2 | Cavity | Resident | Insectivores | 84 | 5 | 3 |
| Tachybaptus_ruficollis* | 4 | Water | Full migrant | Piscivores | 183 | 5.5 | 3 |
| Cinclus pallasii* | 1 | Rock | Resident | Insectivores | 95 | 3.5 | 3 |
| Upupa_epops* | 3 | Cavity | Full migrant | Insectivores | 67.5 | 7.5 | 3 |
| Falco_tinnunculus* | 4 | Cavity | Full migrant | Carnivores | 170 | 2.5 | 3 |
| Sturnus_cineraceu* | 2 | Cavity | Resident | Omnivores | 81.5 | 6 | 3 |
| Phylloscopus schwarzi | 3 | Shrub | Full migrant | Omnivores | 12 | 5 | 2 |
| Mergus merganser | 3 | Cavity | Full migrant | Piscivores | 1299 | 10.5 | 2 |
| Charadrius_dubius | 7 | Ground | Full migrant | Herbivores | 39 | 3.5 | 2 |
| Sitta villosa | 1 | Cavity | Resident | Omnivores | 8.5 | 5.5 | 1 |
| Butastur indicus | 4 | Tree | Full migrant | Carnivores | 435.5 | 3.5 | 1 |
| Calliope calliope | 5 | Shrub | Full migrant | Omnivores | 21.5 | 4 | 1 |
| Pandion haliaetus | 5 | Tree | Full migrant | Carnivores | 1050 | 3 | 1 |
| Apus_apus | 8 | Rock | Full migrant | Insectivores | 35 | 3 | 1 |
| Anthus hodgsoni | 5 | Ground | Full migrant | Omnivores | 21.5 | 5 | 1 |
| Dendrocopos leucotos | 2 | Cavity | Resident | Insectivores | 100 | 4.5 | 1 |
| Eurystomus orientalis | 1 | Cavity | Resident | Insectivores | 149.5 | 4 | 1 |
| Zosterops erythropleurus | 1 | Tree | Full migrant | Omnivores | 12.5 | 3 | 1 |
| Horornis diphone | 3 | Shrub | Full migrant | Omnivores | 13.5 | 4.5 | 1 |
| Saxicola torquatus | 5 | Ground | Full migrant | Omnivores | 17.5 | 6.5 | 1 |
| Cuculus canorus | 5 | Tree | Full migrant | Insectivores | 111.5 | 10 | 1 |
| Strix uralensis | 3 | Cavity | Resident | Carnivores | 830 | 3.5 | 1 |
| Monticola gularis | 3 | Shrub | Full migrant | Insectivores | 35 | 5.5 | 1 |
| Ficedula mugimaki | 3 | Tree | Full migrant | Insectivores | 13.5 | 6 | 1 |

**TABLE S3 Phylogenetic conservatism tests for 6 riparian bird functional traits of Changbai Mountains.**

| Trait | Blomberg’s K | P-value |
| --- | --- | --- |
| Habitat specificity | 0.01 | 0.623 |
| Nest site | 0.45 | 0.01 |
| Migrant status | 0.64 | 0.01 |
| Body mass | 0.01 | 0.87 |
| Trophic level | 1.91 | 0.001 |
| Clutch size | 0.10 | 0.108 |


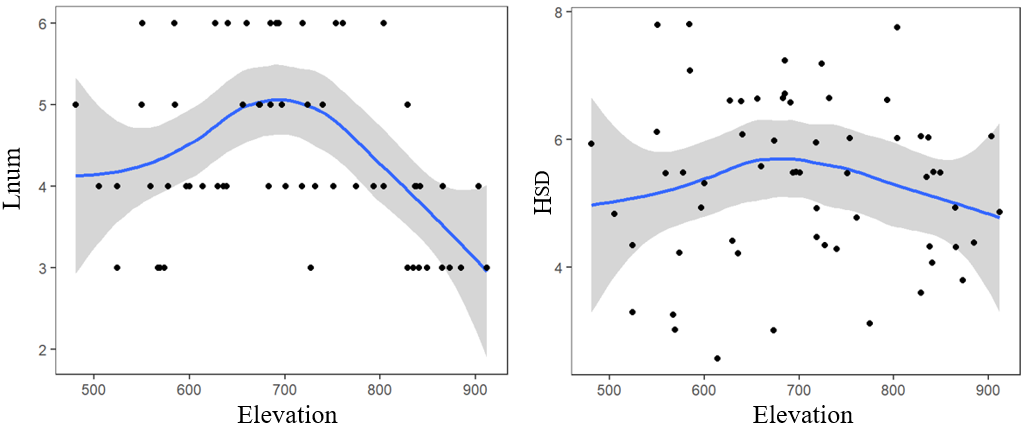


**Figure S1. Distribution patterns of land use types and the standard of the average height of vegetation along longitudinal gradients in studied areas.**


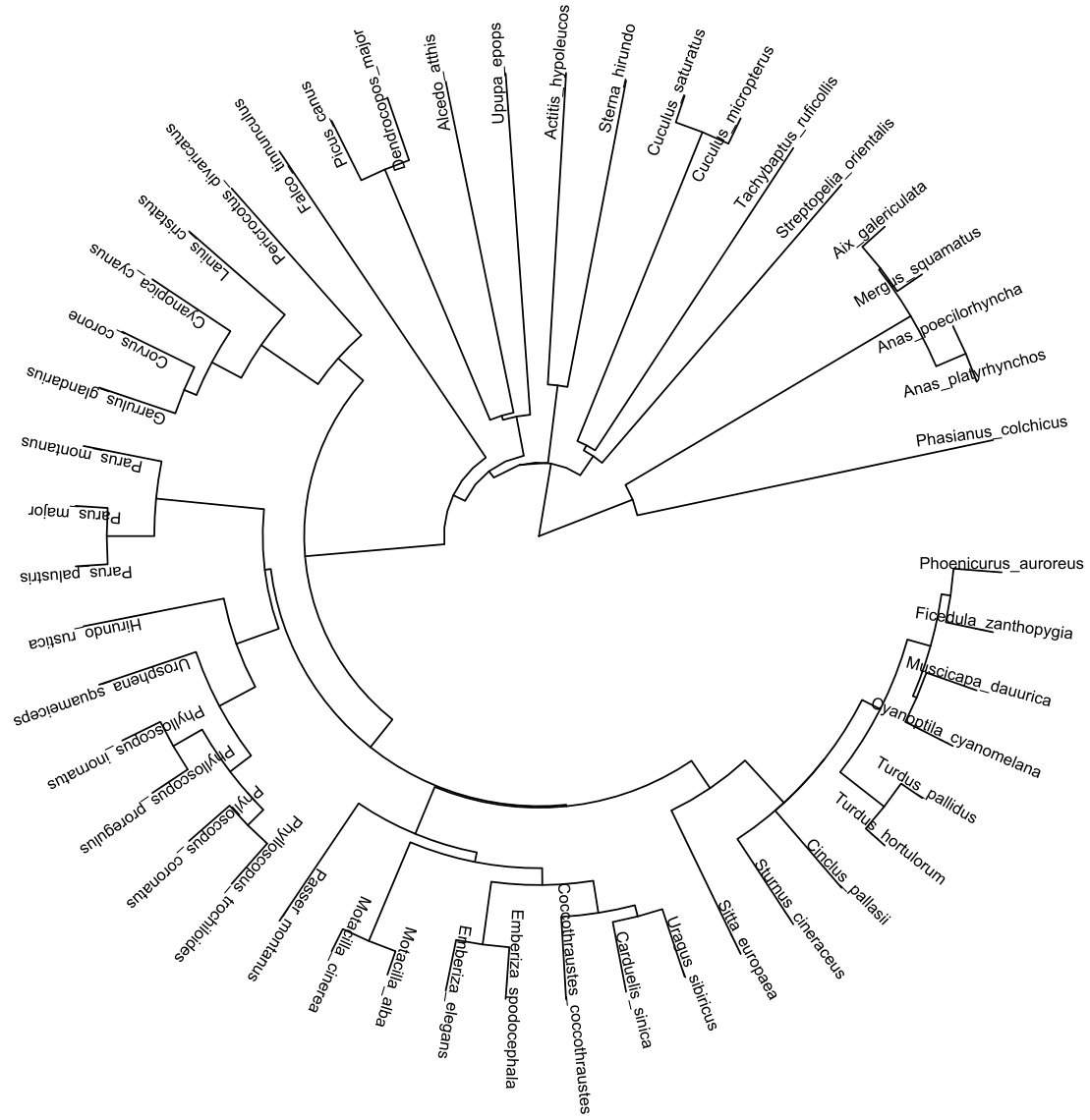


**Figure S2. Visualization of the phylogenetic tree for the 47 bird species included in subsequent analyses.**
